# Effects of SO_2_ on selective catalytic reduction of NO with NH_3_ over a TiO_2_ photocatalyst

**DOI:** 10.1088/1468-6996/16/2/024901

**Published:** 2015-03-10

**Authors:** Akira Yamamoto, Kentaro Teramura, Saburo Hosokawa, Tsunehiro Tanaka

**Affiliations:** 1 Department of Molecular Engineering, Graduate School of Engineering, Kyoto University, Kyotodaigaku Katsura, Nishikyo-ku, Kyoto 615-8510, Japan; 2 Elements Strategy Initiative for Catalysts & Batteries (ESICB), Kyoto University, Kyotodaigaku Katsura, Nishikyo-ku, Kyoto 615-8520, Japan; 3 Precursory Research for Embryonic Science and Technology (PRESTO), Japan Science and Technology Agency (JST), 4-1-8 Honcho, Kawaguchi, Saitama 332-0012, Japan

**Keywords:** photocatalyst, NH_3_-SCR, SO_2_

## Abstract

The effect of SO_2_ gas was investigated on the activity of the photo-assisted selective catalytic reduction of nitrogen monoxide (NO) with ammonia (NH_3_) over a TiO_2_ photocatalyst in the presence of excess oxygen (photo-SCR). The introduction of SO_2_ (300 ppm) greatly decreased the activity of the photo-SCR at 373 K. The increment of the reaction temperature enhanced the resistance to SO_2_ gas, and at 553 K the conversion of NO was stable for at least 300 min of the reaction. X-ray diffraction, FTIR spectroscopy, thermogravimetry and differential thermal analysis, x-ray photoelectron spectroscopy (XPS), elemental analysis and N_2_ adsorption measurement revealed that the ammonium sulfate species were generated after the reaction. There was a strong negative correlation between the deposition amount of the ammonium sulfate species and the specific surface area. Based on the above relationship, we concluded that the deposition of the ammonium sulfate species decreased the specific surface area by plugging the pore structure of the catalyst, and the decrease of the specific surface area resulted in the deactivation of the catalyst.

## 1. Introduction

The emission of nitrogen oxides (NO_*x*_) causes air pollution problems such as acid rain and photochemical smog. Selective catalytic reduction (SCR) of NO_*x*_ with ammonia (NH_3_) is a commercial de-NO_*x*_ process in the stationary and mobile NO_*x*_ emission sources. The main reaction is as follows:
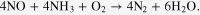



In stationary emission sources, the industrial catalyst for the process is vanadium-oxide-based catalysts such as V_2_O_5_–WO_3_/TiO_2_ [1, 2]. The catalysts show high activity and high selectivity to N_2_ in the temperature window of 523–673 K. This type of catalyst has to be installed upstream to the particulate collector and to the flue-gas desulfurization unit in order to meet the optimum working temperature. However, the catalysts are not available in diesel engines because of the wide temperature window (423–773 K) of the exhaust gas. A promising catalyst which shows high activity at low temperatures is strongly desired for diesel engines.

Mn-based catalysts were eagerly investigated due to their high activity at low temperatures [3–8]. Mn-based catalysts show almost 100% of the conversion in the temperature range of 373–473 K, although the selectivity to N_2_ is slightly lower in some reaction conditions [3, 4]. The problem with Mn-based catalysts is low resistance to SO_2_ poisoning. Kijlstra *et al* [9] proposed that the formation of MnSO_4_ is the main reason for the deactivation of MnO_*x*_/Al_2_O_3_ catalysts. The formed sulfates decomposed at 1020 K, which means regeneration of the catalysts is only possible at much higher temperatures than the reaction temperature. On this point, the simultaneous pursuit of the operation at low temperatures and the high resistance to SO_2_ gas is a challenging and important topic in de-NO_*x*_ technology.

We have previously reported the photo-assisted SCR of NO with NH_3_ over TiO_2_ photocatalysts in the presence of excess oxygen (O_2_) at ambient temperature. In the photocatalytic system, high conversion and high selectivity were achieved at the gas hourly space velocity (GHSV) of 25 000 h^−1^ (conversion of NO > 90%, selectivity to N_2_ > 95%) [10]. We have carried out various investigations: the elucidation of the reaction mechanism using spectroscopic methods [11] and kinetic analysis [12], the improvement of the activity by metal doping [13] and combining of the temperature effect with the photo-SCR [14], and the development of a visible-light-sensitive photocatalyst with dye-sensitization [15]. Other research groups also carried out a theoretical study [16], which verified the proposed reaction mechanism by our group [11, 17], and a kinetic study using an annular fixed-film photoreactor [18]. However, the potential of the photocatalyst for the resistance to SO_2_ gas has not been investigated yet, although the resistance is essential to make the photo-SCR system practicable. Thus, our objective is to investigate the effect of SO_2_ gas on the performance of photo-SCR over the TiO_2_ photocatalyst. In addition, the systematic characterization of the catalyst after the reaction was carried out to elucidate the deactivation mechanism of the TiO_2_ photocatalyst.

## 2. Experimental details

### 2.1. Materials

The TiO_2_ (ST-01) powder was purchased from Ishihara Sangyo Kaisha, Ltd. Before use, the TiO_2_ power was hydrated in distilled water for 2 h at 353 K and then evaporated to dryness at 353 K. The powder after the hydration was tabletted (diameter 20 mm) and calcined at 673 K for 3 h in a furnace under a dry air flow. The tablet was granulated using 25 and 50 mesh sieves to obtain the granules with a diameter of 300–600 *μ*m. Ammonium sulfate (wako) and sodium sulfate (wako) were used as reference samples without further purification.

### 2.2. Photocatalytic reaction

Photo-SCR was carried out using a conventional fixed bed flow reactor at an atmospheric pressure in the same way as in our previous reports [14]. A quartz reactor was used for the reaction, and the reactor volume was 0.12 mL (12 × 10 × 1 mm). 110 mg of TiO_2_ granules with a diameter of 300–600 *μ*m were introduced to the reactor and pretreated at 673 K in a 10% O_2_/He gas at a flow rate of 50 mL min^−1^ for 60 min. The reaction gas composition was as follows: NO (1000 ppm), NH_3_ (1000 ppm), O_2_ (2%), SO_2_ (300 ppm, if present), He balance. A 200 W Hg–Xe lamp equipped with fiber optics, collective lens and a mirror (San-Ei Electric Co., Ltd, UVF-204 S type B) was used as a light source. The measured light irradiance was 360 mW cm^−2^. N_2_ and N_2_O products were analyzed by a SHIMADSU GC-8A TCD gas chromatograph with MS-5A and Porapak Q columns, respectively.

### 2.3. Characterization

The crystalline phase of the TiO_2_ powder was determined by the x-ray diffraction (XRD) technique using a Rigaku Ultima IV x-ray diffractometer with Cu-K*α* radiation (*λ* = 1.5406 Å). The crystallite size was determined from the full width at half maximum (FWHM) of the diffraction peak of the anatase TiO_2_ (101) plane (2*θ* = 25.2°) using the Scherrer equation. The Fourier transform infrared (FTIR) transmission spectra were recorded on a JASCO FT/IR-4200 spectrometer at room temperature at a spectral resolution of 4 cm^−1^, accumulating 16 scans. The background spectrum was measured without any sample in air and was subtracted from the sample spectra. The catalysts before and after the reaction and the reference samples (ammonium sulfate and sodium sulfate) were diluted with KBr by the sample-to-KBr ratio of 1.5:98.5 and 0.2:99.8 (w/w), respectively, and then pressed into pellets. Thermo-gravimetric (TG) analysis and differential thermal analysis (DTA) were performed on a Rigaku Thermo plus TG 8120 apparatus at a heating rate of 5 K min^−1^ under a dry air flow condition at a flow rate of 80 mL min^−1^ in the range of 298–1173 K using Al_2_O_3_ pans. The XPS measurement was conducted on a Shimadzu ESCA-3400 spectrometer. Samples were mounted on a silver sample holder using a conductive carbon tape and were analyzed using Mg K*α* radiation in a vacuum chamber in 0.1 eV steps. The position of the carbon peak (284.6 eV) for C1s was used to calibrate the binding energy for all the samples. The surface composition was estimated by the band areas of the XP spectrum of S2p, N1s, Ti2p and O1s and the corresponding relative sensitivity factors [19]. Elemental analyses (EA) were performed on two CHN analyzers (MT-5, Yanaco Co., Ltd and JM10, J-Science Lab Co., Ltd) to analyze the contents of C, H and N, and combustion ion chromatography (Dionex ICS-1500, Mitsubishi Chemical Analytech AQF-2100H) was used to analyze the S content. The N_2_ adsorption/desorption isotherm was measured at 77 K using liquid nitrogen. The Brunauer–Emmett–Teller (BET) method was utilized to calculate the specific surface areas (*S*_BET_). The total specific surface area (*S*_tot_) and the external specific surface area (*S*_ext_) were calculated from the linear fitting of the *V*–*t* plots (fitting range: 0–0.6 nm for the *S*_tot_ and 3.6–4.6 nm for the *S*_ext_). The internal specific surface (*S*_int_) area for the mesoporous materials was obtained by subtracting the *S*_ext_ from the *S*_tot_. The pore-sized distribution was calculated from the Barrett–Joyner–Halenda (BJH) plots.

## 3. Results and discussion

### 3.1. Effect of SO_2_ addition on the activity of the photo-SCR

**Figure 1.**
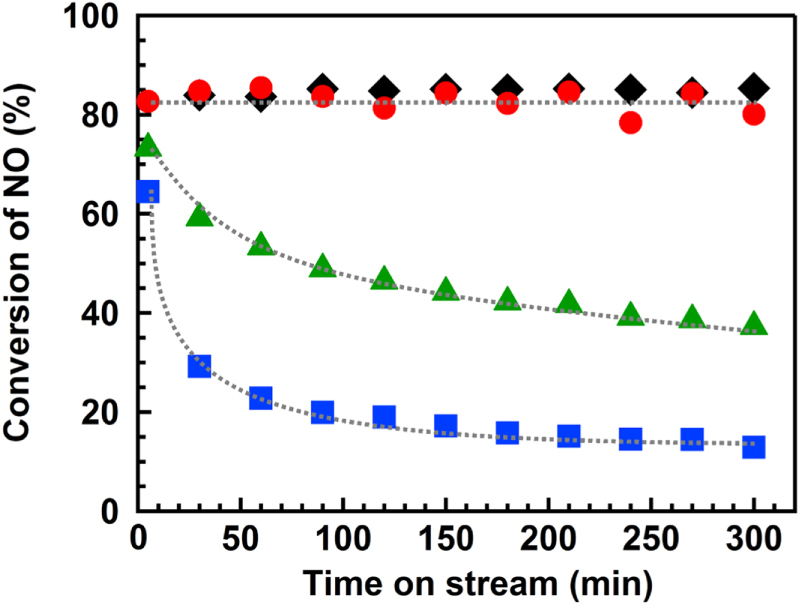
Time course of the photo-SCR in the presence or absence of SO_2_ gas at various temperatures. (◆) SO_2_: 0 ppm, 433 K, (▲) SO_2_: 300 ppm, 373 K, (■) SO_2_: 300 ppm, 433 K, (●) SO_2_: 300 ppm, 553 K. NO: 1000 ppm, NH_3_: 1000 ppm, O_2_: 2%, He: balance gas, flow rate: 200 mL min^−1^, GHSV: 100 000 h^−1^, light source: 200 W Hg–Xe lamp.

Figure 1 shows the time course of the photo-SCR over the TiO_2_ photocatalyst under illumination in the presence or absence of SO_2_ (300 ppm) gas at various temperatures. The conversion of NO was stable for 300 min at 433 K in the absence of SO_2_ gas, as we reported previously [14]. In the presence of SO_2_ at 433 K, the conversion of NO decreased with the reaction time, which indicates that the SO_2_ poisoned the catalyst, as in the case of the Mn-based catalysts [9]. The reaction temperature had a significant effect on the deactivation rate of the TiO_2_ photocatalyst. At 373 K, the conversion decreased more rapidly than at 433 K, and the conversion was almost stable for at least 300 min at 553 K. For simplification, the sample before the reaction was abbreviated as BR, and the samples after the reactions at 373 K, 433 K and 553 K were abbreivated as AR-373K, AR-433K and AR-553K, respectively.

### 3.2. XRD patterns

**Figure 2.**
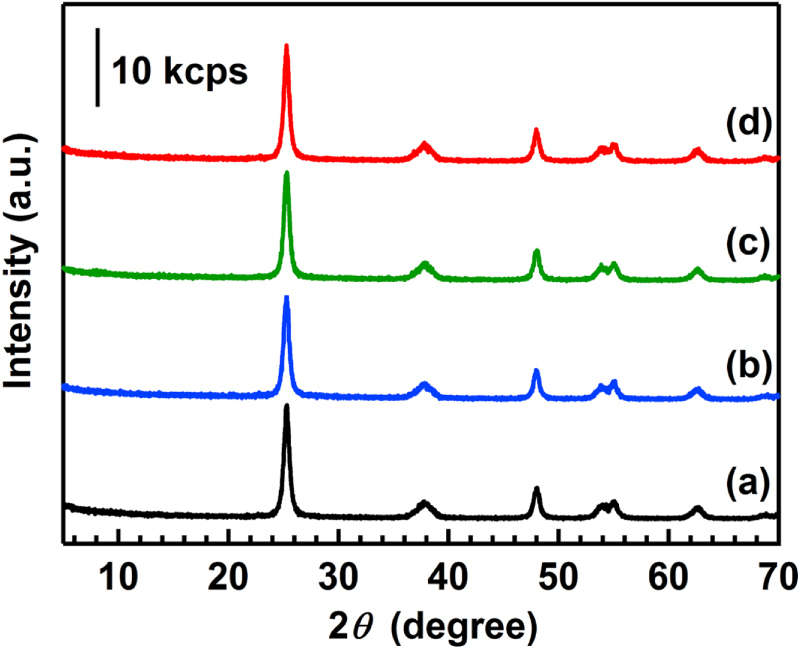
XRD patterns of the catalysts before and after the reaction. (a) BR, (b) AR-373K, (c) AR-433K and (d) AR-533K. Patterns are offset for clarity.

The XRD patterns of the catalysts are shown in figure 2. In all the catalysts, only the diffraction pattern of anatase TiO_2_ was observed. Crystalline sizes of anatase TiO_2_ were estimated by the Scherrer equation using the diffraction peaks of (101), and the results are listed in table 1. The crystalline size did not change (about 15 nm) after the reaction, revealing that the aggregation of TiO_2_ particles did not occur under the reaction conditions at all the reaction temperatures.

**Table 1.** Crystalline size and specific surface area of catalysts.

Sample	*d*^a^ (nm)	*S*_BET_^b^ (m^2^ g^−1^)	*S*_tot_^c^ (m^2^ g^−1^)	*S*_ext_^d^ (m^2^ g^−1^)	*S*_int_^e^ (m^2^ g^−1^)
BR	15.2	121	123	10.6	112
AR-373K	15.0	84.3	87.0	10.8	76.2
AR-433K	15.2	96.6	99.6	9.0	90.6
AR-553K	15.3	115	118	9.0	109

a Crystalline size calculated from the FWHM of the diffraction peak of the anatase TiO_2_ (101) plane.

b Specific surface area determined by N_2_ adsorption isotherm at 77 K using the BET method.

c Total specific surface area calculated from the *V*–*t* plots.

d External specific surface area calculated from the *V*–*t* plots.

e Internal specific surface area calculated by subtracting the external specific surface area from the total specific surface area.

### 3.3. N_2_ adsorption/desorption experiments

In N_2_ adsorption/desorption experiments at 77 K (figure S1), all the samples exhibited a typical IV-type isotherm and had a vertically long hysteresis loop in the relative pressure (*P*/*P*_0_) range of 0.8–1.0, suggesting that all the samples had a porous structure. The pore-sized distribution of the catalyst before the reaction had a sharp peak at 10 nm (figure S2(A)), and the size was the same order of the crystalline size of TiO_2_ (15 nm). These results indicate that the mesopores were formed by the gaps between primary TiO_2_ particles. The peak became smaller after the reaction in the presence of SO_2_ gas. The specific surface area calculated from the *V*–*t* plots (figure S3(B)) is summarized in table 1. The *S*_tot_ calculated from the *V*–*t* plots was in good agreement in the *S*_BET_. The *S*_ext_ of all the samples was estimated from the *V*–*t* plots as 9.0–10.8 m^2^ g^−1^. The *S*_int_ was calculated by subtracting the *S*_ext_ from the *S*_tot_. The *S*_int_ was 76.2–112 m^2^ g^−1^, which shows that the largest part of the *S*_tot_ was derived from the mesopores.

### 3.4. FTIR spectra

**Figure 3.**
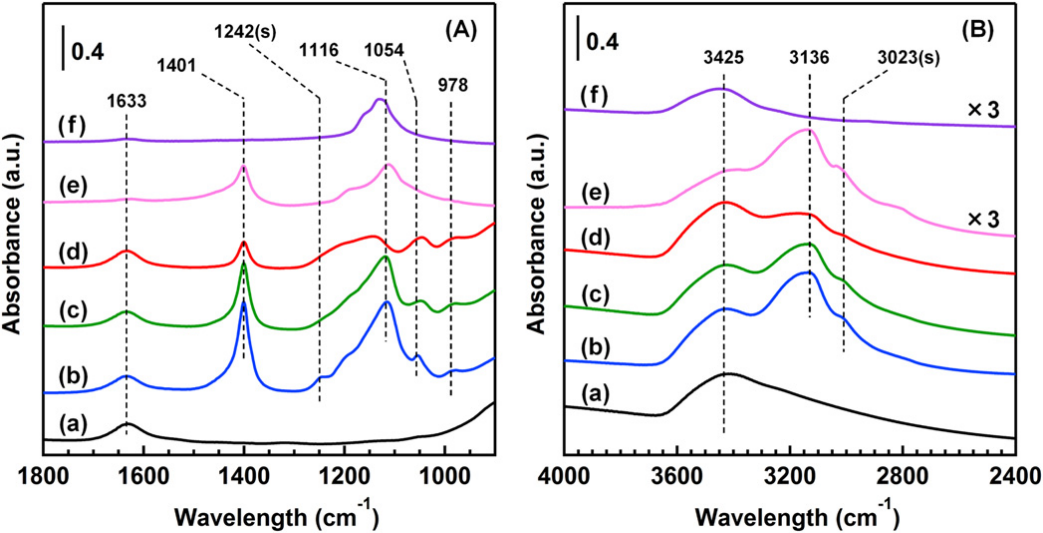
FTIR spectra of the catalysts and the reference samples in the region of (A) 900–1800 cm^−1^ and (B) 2400–4000 cm^−1^. (a) BR, (b) AR-373K, (c) AR-433K, (d) AR-533K, (e) (NH_4_)_2_SO_4_ and (f) Na_2_SO_4_. Spectra are offset for clarity.

Figure 3(A) shows the FTIR spectra of the catalysts in the region of 900–1800 cm^−1^. In all the catalysts, a band at 1633 cm^−1^ was observed and was attributed to the deformation vibration of water molecules adsorbed on the TiO_2_ surface. New bands at 1401, 1242, 1116, 1054 and 978 cm^−1^ appeared after the reaction in the presence of SO_2_ at 373 K. The sharp band at 1401 cm^−1^ was assigned to the bending vibration of ammonium (NH_4_^+^) ions [20] and was also observed in the case of the reference (NH_4_)_2_SO_4_ powder. Free sulfate ions (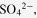 *T*_d_ symmetry) show two infrared peaks at 1104 (*ν*_3_) and 613 (*ν*_4_) cm^−1^ [20]. The band at 1116 cm^−1^ was due to *ν*_3_ vibration of free sulfate ions and was also observed in both the cases of (NH_4_)_2_SO_4_ and Na_2_SO_4_. When a 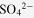 ion is bound to the TiO_2_ surface, the symmetry can be lowered to either *C*_3v_ or *C*_2v_. The lowering symmetry causes the split of the *ν*_3_ vibration band into two peaks for a *C*_3v_ symmetry and splits into three peaks for a *C*_2v_ symmetry [20]. Thus, the bands at 1242, 1054 and 978 cm^−1^ are assigned to the surface-coordinated 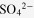 ions. The surface-coordinated 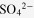 ions could have the *C*_2v_ symmetry based on the number of bands. The 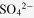 ion with a *C*_2v_ configuration is either chelating bidentate or bridge bidentate [20]. In AR-433K, the shape of the spectrum was similar to that of AR-373K, although the absorbance of the bands at 1401 and 1116 cm^−1^ were slightly weaker than those of AR-373K. In AR-553K, the band at 1116 cm^−1^ disappeared, which means that the deposition of free sulfates was inhibited in the reaction at 553 K, although the other bands at 1401, 1054 and 978 cm^−1^ remained. In the region of 2400–4000 cm^−1^ (figure 3(B)), three adsorption bands at 3425, 3136 and 3023 cm^−1^ were observed after the reaction at 373 K. The broad band between 3600–2800 cm^−1^ is the stretching vibration of OH groups derived from surface hydroxyl groups and adsorbed water molecules, which also appeared in the sample before the reaction. The other two bands were observed in the catalysts after the reaction. The bands at 3136 and 3023 cm^−1^ are attributed to the asymmetric stretching vibration (*ν*_3_) and symmetric stretching vibration (*ν*_1_) of the NH_4_^+^ ions, respectively. The two bands also appeared in the case of (NH_4_)_2_SO_4_, which strongly advocated the generation of NH_4_^+^ ions after the reaction. The peak intensity at 3136 and 3023 cm^−1^ decreased as the reaction temperature increased, which was consistent with the decrease of the band at 1401 cm^−1^ in figure 3(A). The FTIR results clearly revealed the generation of the free and surface-coordinated 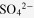 ions and NH_4_^+^ ions on the TiO_2_ surface after the reaction. The conversion of NO after 300 min of the reaction decreased in the following order: AR-553K (80.1%) > AR-433K (37.2%) > AR-373K (12.9%). The order was consistent with that of the peak intensities of the free 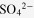 ions and NH_4_^+^ ions, which suggests that the generation of ammonium sulfate species (e.g. (NH_4_)_2_SO_4_ and (NH_4_)HSO_4_) induced the deactivation of the catalyst.

### 3.5. TG-DTA analysis

**Figure 4.**
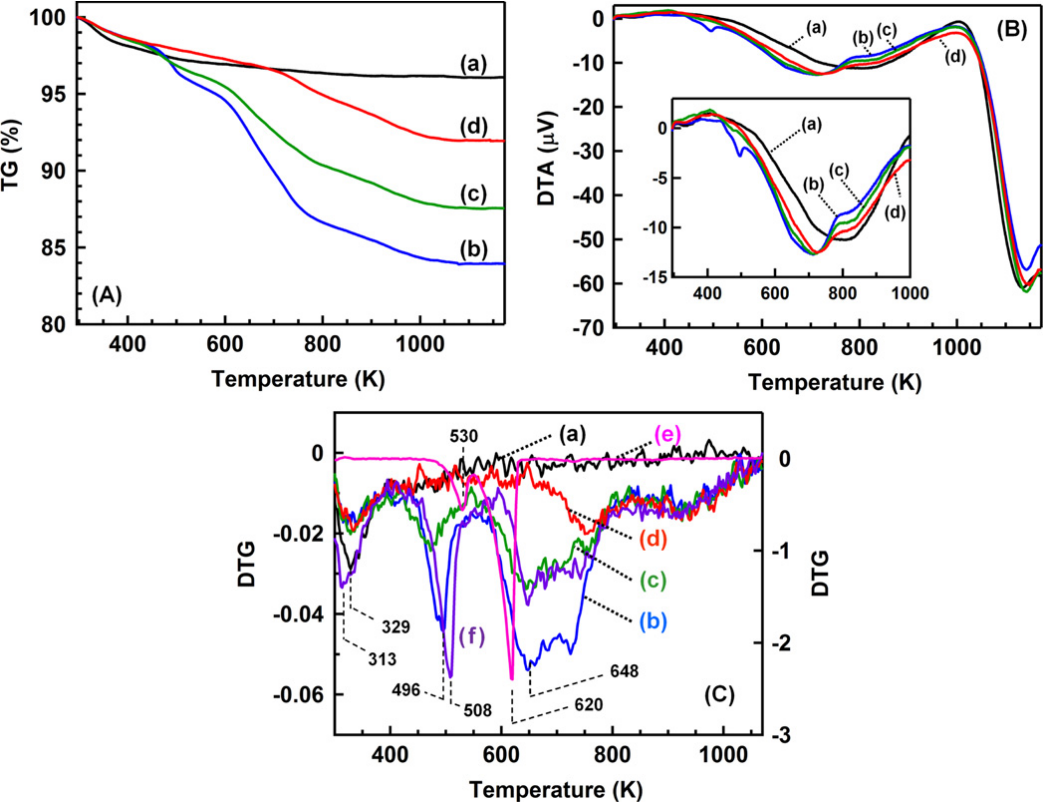
(A) TG profiles, (B) DTA profiles and (C) derivative thermogravimetry (DTG) profiles of the catalysts and the reference samples. (a) BR, (b) AR-373K, (c) AR-433K, (d) AR-533K, (e) (NH_4_)_2_SO_4_ and (f) physical mixture of (NH_4_)_2_SO_4_ and TiO_2_ ((NH_4_)_2_SO_4_: 10% by weight).

TG profiles of the catalysts are shown in figure 4(A). Several steps of the weight loss were observed in all the catalysts after the reaction, although the TiO_2_ has only one step of the weight loss around 330 K. From room temperature to 1173 K, the weights of BR, AR-373K, AR-433K and AR-553K decreased by 3.9, 16.1, 12.5 and 8.1%, respectively. Figure 4(B) shows the DTA profiles of the catalysts. All the profiles had a strong exothermic band around 1150 K without the weight loss, which was derived from the phase transition of TiO_2_ from anatase to rutile. In the DTA profile of BR (see the inset of figure 4(B)), a broad exothermic band was observed around 820 K without the weight loss. The peak was observed in all the catalysts before and after the reaction and was possibly due to the crystallization of TiO_2_ [21]. In addition, other exothermic bands were observed around 500 and 700 K in the case of the catalysts after the reaction.

Figure 4(C) shows the first derivatives of the TG profiles (DTG) in figure 4(A). The negative band around 330 K was observed in all the catalysts and was assigned to desorption of molecular water. Other weight loss peaks were observed around 496 K, 648 K, 740 and 940 K. DTG profiles of the (NH_4_)_2_SO_4_ powder and a physical mixture sample of the (NH_4_)_2_SO_4_ powder and the TiO_2_ powder are also shown in figure 4(C). In the (NH_4_)_2_SO_4_ powder, two peaks were observed at 530 K and 620 K, which were attributed to the following reactions: (1) and (2), respectively [22]
1
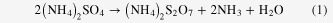

2
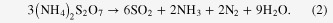



In the physical mixture sample of the (NH_4_)_2_SO_4_ powder and the TiO_2_ powder, the weight loss profile had negative peaks around 313 K, 508 K, 648 K, 760 K and 940 K, and the peak positions were in good agreement with those of the profile of AR-373 K. The analogy of the profiles strongly supports the generation of the (NH_4_)_2_SO_4_ species on the TiO_2_ surface after the reaction at 373 K. The bands around 500 K and 650 K decreased as the reaction temperature increased from 373 K to 553 K, which suggests that the increase of the reaction temperature inhibits the deposition of the (NH_4_)_2_SO_4_ species on the TiO_2_ surface.

### 3.6. XPS

**Figure 5.**
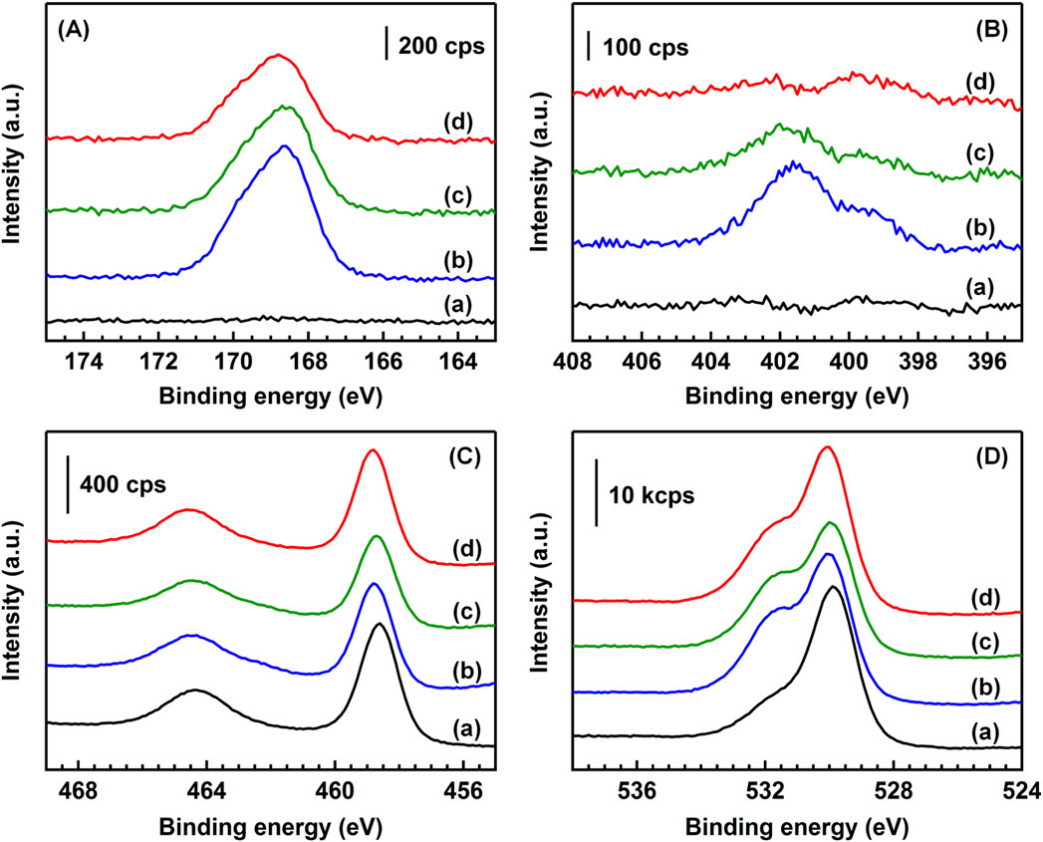
XP spectra of (A) S 2p, (B) N 1s, (C) Ti 2p and (D) O 1s of the catalysts. (a) BR, (b) AR-373K, (c) AR-433K and (d) AR-533K. Patterns are offset for clarity.

In the S 2p XP spectra of BR (figure 5(A)), no band was observed in the range of 165–175 eV. In all the catalysts after the reaction, asymmetric bands were observed at the peak position of 168.6–168.8 eV. The asymmetry of the bands is because of the overlap of the split sublevels of the 2p_3/2_ and 2p_1/2_ states of S atoms (separation of bands: 1.2 eV) by spin–orbit coupling [23]. The spectra of the catalysts after the reaction were reasonably fitted using two Gaussian functions (figure S3 in the supplementary data and table S1). In all the catalysts after the reaction, the peak positions were 168.5–168.6 and 169.7–169.9 eV, which corresponded to the peak positions of S 2p_3/2_ and S 2p_1/2_ of the sulfate (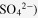 species, respectively [20]. The ratios of the peak areas of S 2p_3/2_ to those of S 2p_1/2_ were 1.96–1.99 in all the catalysts after the reaction, and the values were in good agreement with the theoretical value of 2. The FWHM of the each peak was the same in all the catalysts (about 1.6 eV), which suggests that the S 2p XP spectra of the catalysts are derived from a single sulfur species of 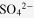 (S^6+^).

The N 1s XP spectra are shown in figure 5(B). The peak positions of the N bands of the NO_2_^−^ and NO_3_^−^ species on the TiO_2_ surface were reported to be 403.5 eV and 407.0 eV, respectively [24, 25]. There was no band in the region of 403.5–407.0 eV, indicating that the NO_2_^−^ and NO_3_^−^ species did not exist in all the catalysts after the reaction. In AR-373K, there were two bands with the peak positions at 399.9 and 401.8 eV. The shoulder band at 399.9 eV was attributed to N atoms of NH_3_ adsorbed on Lewis acid sites of TiO_2_, and the band at 401.8 eV was attributed to N atoms of the NH_4_^+^ species adsorbed on Br⊘nsted acid sites and/or ammonium salts [20]. The band at 401.8 eV decreased with increasing the reaction temperature and disappeared in the spectrum of AR-533K.

In the Ti 2p XP spectra (figure 5(C)), the binding energies of the band of Ti 2p_1/2_ and 2p_3/2_ were 458.6–558.8 and 464.4–464.6 eV in all the catalysts before and after the reaction (table S2 in the supplementary data). The values were consistent with those reported in the literature [20]. The O 1s XPS bands (figure 5(D)) of BR were fitted by two Gaussian functions. The peak positions of the two bands were 530.9 eV and 529.8 eV. The band with a higher binding energy of 530.9 eV was due to the O atom of water molecules adsorbed on the surface and surface hydroxyl groups, and the band at 529.8 eV corresponded to the lattice oxygen on the TiO_2_ surface [26]. The spectra of the samples after the reaction were fitted using two Gaussians. The result of the fitting is shown in table S3. The relative intensities of the higher energy band to the lower energy band increased after the reaction, which was interpreted by the generation of sulfate species because the oxygen atoms in sulfate have a band at 532.4 eV, as shown in figure S4 in the supplementary data.

Table 2 shows the surface composition estimated by the XPS analysis. The ratio of S atoms to Ti atoms (S/Ti) decreased in the order of AR-373K > AR-433K > AR-553K. The N atoms to Ti atoms (N/Ti) ratio also decreased with increasing the reaction temperature. The decreases of the S/Ti and N/Ti ratio should be mainly due to the decomposition of the (NH_4_)_2_SO_4_ species based on the FTIR and TG-DTA analyses. The ratio of N atoms to S atoms (N/S) decreased from 0.41 (373 K) to 0.10 (553 K) with the reaction temperature. The N/S values of the catalysts were lower than that of (NH_4_)_2_SO_4_ powder (0.85). The low N/S values of the catalysts suggests the existence of sulfur species other than (NH_4_)_2_SO_4_. From the XPS analysis, SO_3_^2−^ species were not detected, and the valence of all the sulfur species was +6. The FTIR spectroscopy revealed the generation of the surface-coordinated 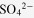 species with the *C*_2v_ and/or *C*_3v_ symmetries. Thus, the low N/S values were due to the generation of the surface-coordinated 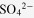 species. Based on the above discussion, the decrease of the N/S values with the increase of the reaction temperature is interpreted by the preferential decrease of the (NH_4_)_2_SO_4_ species compared to the surface-coordinated 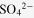 species with increasing the reaction temperature.

**Table 2.** Surface composition estimated by XPS.

Sample	S/Ti	N/Ti	N/S
BR	0	0	—
AR-373K	0.28	0.11	0.41
AR-433K	0.27	0.07	0.26
AR-553K	0.14	0.01	0.10
(NH_4_)_2_SO_4_	—	—	0.85

### 3.7. Elemental analysis (EA)

Concentrations of sulfur atoms and nitrogen atoms were estimated by EA (table 3). The concentration of S atoms and N atoms increased after the reaction and decreased with increasing the reaction temperature. The tendency corresponded to the results of XPS analysis. Surface densities of each atom were calculated by dividing the concentrations by the BET specific surface area of BR (table 1). The surface density of the Ti atoms was calculated to be 7.0 nm^−2^ using a (100) plane of anatase TiO_2_ [27] and the BET specific surface area of BR. In AR-373K, the surface densities of sulfur atoms and nitrogen atoms were 5.5 and 9.3 nm^−2^, respectively, and the sum of the values were almost twice as much as that of Ti atoms. The result suggests the generation of a bulk (NH_4_)_2_SO_4_ species. The N/S ratio estimated by EA (table 3) had the same tendency as that evaluated by XPS: the N/S ratio decreases with increasing the reaction temperature. In AR-373K, the N/S ratio was 1.7 and was close to 2, which was the theoretical value of the chemical composition of (NH_4_)_2_SO_4_. Thus, the biggest part of the 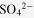 species is present as a (NH_4_)_2_SO_4_ salt in AR-373K. However, the N/S ratio by the XPS analysis is 0.41, which was almost half of the experimental value of the reference (NH_4_)_2_SO_4_ powder (table 2). The discrepancy between N/S ratios estimated by EA and XPS could be interpreted by considering the generation of the bulk (NH_4_)_2_SO_4_ species. XPS analysis is more sensitive to surface-coordinated 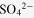 species than bulk (NH_4_)_2_SO_4_ species. The N/S ratio estimated by XPS should be lower than the real amount of the (NH_4_)_2_SO_4_ species, which was estimated by the EA, when the (NH_4_)_2_SO_4_ species has a bulk structure. Thus, the lower N/S ratio by XPS than that by EA also implies the generation of the bulk (NH_4_)_2_SO_4_ species.

**Table 3.** Results of elemental analysis.

	Concentration (wt%)	Surface density (nm^−2^)	N/S
Sample	S	N	S	N	(atom/atom)
BR	0.06	0	0.09	0	0
AR-373K	3.5	2.6	5.5	9.3	1.7
AR-433K	2.6	1.6	4.1	5.6	1.4
AR-553K	1.7	0.64	2.7	2.3	0.85

### 3.8. Structure of the ammonium sulfate species and deactivation mechanism

**Figure 6.**
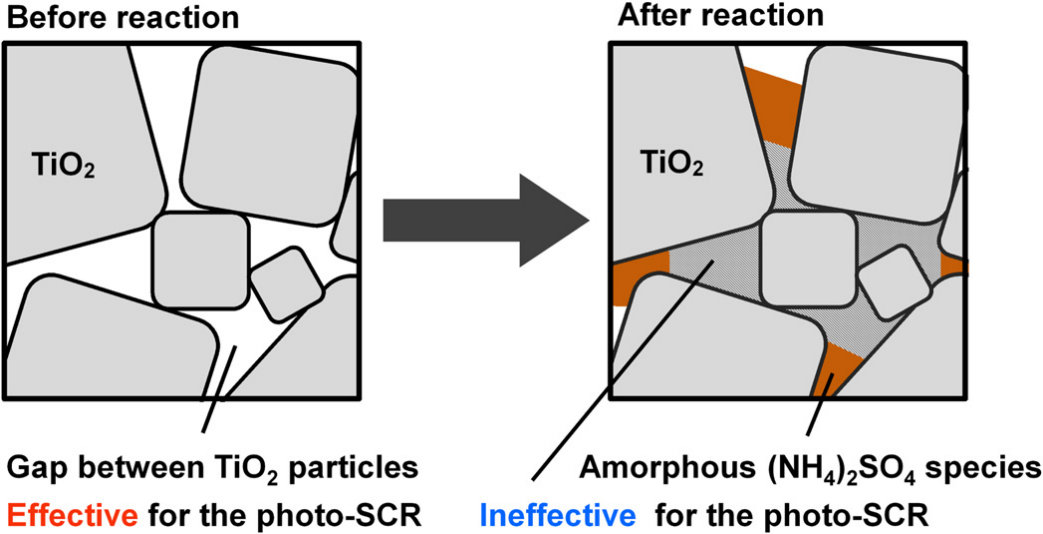
Deposition model of sulfate species of the catalysts.

N_2_ adsorption/desorption experiments revealed the decrease of the *S*_tot_ after the reaction, which was mainly due to the decrease of the *S*_int_. The decrease of the *S*_int_ is not due to the aggregation of the TiO_2_ particles during the reaction because the crystalline size of the TiO_2_ particles did not change after the reaction (table 1). FTIR, TG-DTA, XPS and EA revealed the generation of the bulk (NH_4_)_2_SO_4_ species on the TiO_2_ surface after the reaction. The amount of (NH_4_)_2_SO_4_ in AR-373K, which contains the largest amount of S and N atoms among the three catalysts after the reaction, is calculated to be 12 wt% assuming that all the N atoms, which were estimated by EA, exist in the (NH_4_)_2_SO_4_ form. However, the XRD diffraction peak of the bulk (NH_4_)_2_SO_4_ species was not observed in AR-373K, which implies that the generated bulk (NH_4_)_2_SO_4_ species has an amorphous structure. Thus, the generated bulk (NH_4_)_2_SO_4_ species plugged the pores of the catalysts, which resulted in the decrease of the *S*_int_ (figure 6).

**Figure 7.**
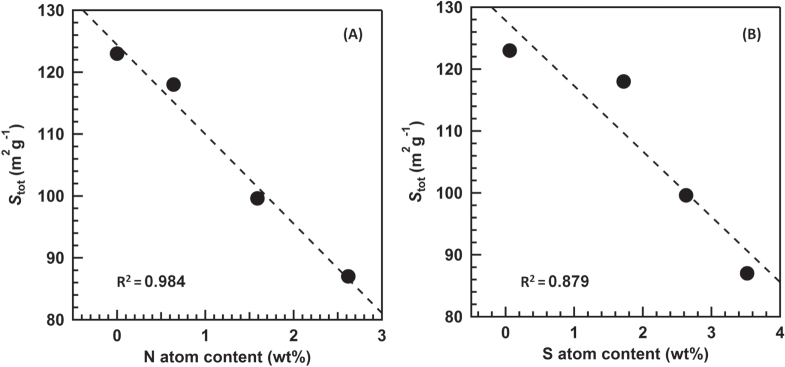
Effect of the contents of N atoms (A) and S atoms (B) on the *S*_tot_ estimated from the *V*–*t* plots.

**Figure 8.**
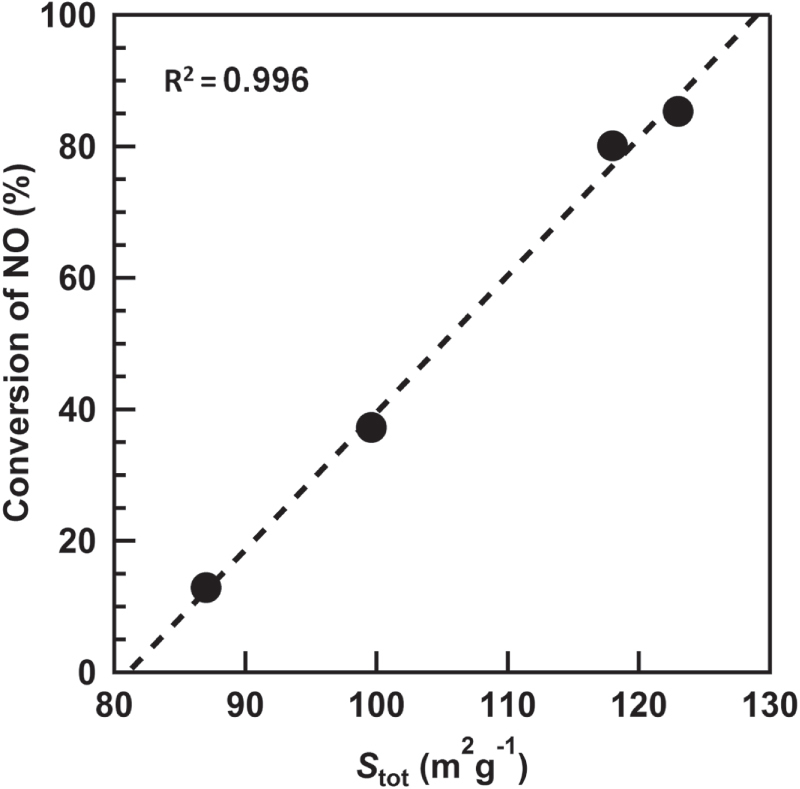
Effect of the *S*_tot_ estimated by the *V*–*t* plots on the conversion of NO after 300 min of the photo-SCR at various temperatures.

The amount of the (NH_4_)_2_SO_4_ species decreased with increasing the reaction temperature. Figures 7(A) and (B) show a correlation between the *S*_tot_ and the contents of N and S estimated by the EA. The increment of the contents of S and N drastically decreased the *S*_tot_. FTIR analysis revealed two 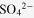 species: one is the (NH_4_)_2_SO_4_ species and the other is the surface-coordinated 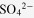 species. The negative linear correlation for the N content strongly suggests that the decrease of the *S*_tot_ is not because of the generation of the surface-coordinated 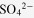 species but is because of the bulk (NH_4_)_2_SO_4_ species. The conversion of NO after 300 min of the reaction was plotted vs. the *S*_tot_ (figure 8). The strong positive and linear correlation was obtained, which indicates that the decrease of the *S*_tot_ results in the decrease of the conversion of NO. Based on the above discussion, we concluded that the generation of the (NH_4_)_2_SO_4_ species plugged a part of the mesopores derived from the gap between the TiO_2_ particles, which resulted in the decrease of the *S*_tot_ and the deactivation of the catalyst.

## 4. Conclusions

In this study, tolerance to SO_2_ gas was investigated in the photo-SCR of NO with NH_3_ over the TiO_2_ photocatalyst. The introduction of SO_2_ drastically decreased the conversion of NO at 373 K. The increment of the reaction temperature drastically improved the stability of the catalyst, and at 553 K, the deactivation was not observed for 300 min of the reaction. FTIR and XPS results suggest that two 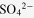 species exist on the TiO_2_ surface: the surface-coordinated 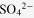 species and the free 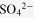 species as a bulk (NH_4_)_2_SO_4_ formed after the photocatalytic reaction. The deactivation occurs due to a pore plugging by the deposition of the (NH_4_)_2_SO_4_ species on the TiO_2_ surface on the basis of the correlation among the contents of N and S atoms after reaction, the *S*_tot_ estimated by *V*–*t* plots and the photocatalytic activity.
